# Editorial

**Published:** 1868-08

**Authors:** 


### Editorial.

Medical College Instruction.—Most, and perhaps all, the medical schools in our country have issued their announcements for the annual courses of instruction for 1868-9. With very few exceptions, they indicats a continuance of the same heterogeneous courses of instruction that have characterized our medical collegee ever since the colonial period of our country's history. The facilities for clinical teaching, in most of the schools, have been greatly increased during the last few years, and many of them have increased the number of their faculties and extended their lecture-terms to five months. But with only one exception, so far as we have observed, they still cling to the old, and often condemned, method of teaching all their students in one class, and subjecting them to the formal task of listening to lectures on all the departments of medical science and art in the space of four or five months. There is, however, one exception.

The Chicago Medical College, founded in 1859, and organized on the basis of systematic and progressive teaching, has this year completed its curriculum in accordance with the plan of its founders, and in full accordance with the recommendations of the Convention of Medical College Delegates, in Cincinnati, in 1867, and with the repeated recommendations of the American Medical Association. With a full corps of professors; with a complete curriculum, embracing every department of medical science and art, as it exists at this time; with the most ample facilities for hospital clinical instruction; and every means required for demonstrative teaching in all the departments, this college offers to the profession every facility for a systematic and complete medical education.

Having divided the whole number of branches taught into three groups, corresponding with each of the three years required for medical study, and extended the regular annual lecture-term to six months, the intelligent student can here enter upon the important work of acquiring a knowledge of medicine, in the same rational and systematic manner that he would in studying any other departments of science. Instead of crowding all into one class, those commencing study can limit their attention to such branches only as are adapted to their first year of pupilage, and thereby lay a proper foundation. Those in the middle part of their period of study can limit their attention to just the branches adapted to the second year; and the same with the third. Daily examinations in the lecture-room, and thorough examinations at the close of each annual course, complete the work of securing to the student both completeness of knowledge and thoroughness of mental discipline.

The Chicago Medical College thus presents to the profession a theoretical completeness of organization, but its actual practical advantages are equally perfect. In clinical advantages it has no superior in this or any other country. Mercy Hospital, founded in 1850, so far as medical and surgical attendance and clinical instruction are concerned, is as much a part of Chicago Medical College, as Bellevue Hospital is of Bellevue Hospital Medical College in New York. This hospital, with its medical, surgical, and lying-in departments, not only constitutes an excellent clinical school, but the classes in attendance are always so divided that each student is enabled to receive direct personal instruction in all the methods of examination and diagnosis. Cook County Hospital, always filled with a large number and variety of patients, is only five minutes' walk from the college building. It is supplied with an able corps of clinical teachers, among whom are Professors H. A. Johnson and Tho's Bevan, of the Chicago Medical College Faculty.

These two hospitals not only afford the most ample field for the clinical study of practical medicine, surgery, obstetrics, and morbid anatomy, but the wards for diseases of the eye and ear in the County Hospital, under the charge of Dr. J. S. Hildreth, afford full and thorough clinical instruction concerning the diseases and accidents of these important organs. Full personal instruction of the student in the use of the ophthalmoscope, laryngoscope, endoscope, microscope, and the various means of physical exploration and diagnosis, is included as a part of the ordinary courses of instruction by the members of the faculty, without extra charge. In all the schools of New York, Philadelphia, or Boston, if the student obtains direct personal instruction and practice in auscultation, ophthalmology, microscopy, etc., he must pay some professor or assistant an extra fee of from $15 to $30, for just what he receives fully in the regular course in the Chicago Medical College.

We are thus particular in calling attention to this college, not because we happen to hold a place in its faculty, but simply for the reason that the degree of patronage it receives from the profession of the North-west will have an important bearing on the success of the movement for a more extended and rational system of medical college instruction throughout the whole country.

For more than a-quarter of a century, the profession, through the National and State organizations, have been calling upon the colleges for the adoption of a system of instruction more adequate to the existing state of medical science, and better adapted to secure a thorough education of those who shall enter the ranks of the profession. These demands finally culminated in the holding of a convention of delegates from medical colleges exclusively, in Cincinnati, in May, 1867. That convention, after full consideration, very unanimously recommended a revised system of medical college instruction, embracing a standard of preliminary education, six months' lecture-term, division of the various branches into three groups, and the requirement of three courses of lectures for graduation, etc. The plan received the immediate and unanimous approval of a full meeting of the American Medical Association, and, within the last few months, it has been equally sanctioned and its adoption urged upon the colleges by the State Medical Societies of Illinois, Missouri, Iowa, and others.

Chicago Medical College, complete in every department; commanding every facility for instruction that can be found in any part of our country; and pecuniarily free from all embarassment, present or prospective, has boldly and fully adopted the revised system recommended. If those enlightened members of the profession throughout the North-west, who have long desired to see our medical colleges substantially improved, will so direct their influence over students as to cause this school to be well sustained, it will speedily induce others to follow her example, and the long-desired change will become universal. Will each one recognize his personal responsibility in this matter?

---

CONDENSATION.—The Senior Editor of the *Chicago Medical Journal* writes as follows, in reference to his new associate in the editorial work: "He will assume the task of *translations* of valuable papers from foreign medical journals, and in *each No.* furnish an epitome of *all* that attracts professional attention in the Old World."

Just what "*the task of translations*" may mean, we will not stop to inquire. But, inasmuch as "*each No.*" of the *Chicago Medical Journal* contains only 32 pages, and some of these are always devoted to the "*Apostle*," either the professional attention in the Old World must be very inactive, or the Associate-Editor will exhibit an extraordinary power of *epitomizing*.

Sickness of Pregnancy.—A writer in the *London Lancet* suggests belladonna, as probably the best remedy for allaying the reflex or sympathetic gastric disturbance of pregnancy. For several years we have used small doses of belladonna or atropine, before breakfast and dinner, for this purpose, with success. Sometimes, the relief has been more complete when each dose of the narcotic was associated with five or six drops of hydrochloric acid diluted with water.

---

A Contrast.—The Medical School of the University of Berlin has 401 students in attendance. Its corps of instructors consists of 14 ordinary professors, 12 extraordinary, and 24 private instructors. We have several medical schools in this country that claim an attendance of over 400 students, who receive all their instruction annually from 7 or 8 professors, and in the short space of 4 or 5 months. Either the faculty in Berlin must be very old fogyish, or some of our schools are little better than a farce.

---

Professors.—The Chair of Physiology in Jefferson Medical College, made vacant by the resignation of Prof. Robly Dunglison, has been filled by the appointment of J. Atkin Meigs, M.D., of Philadelphia. Tho's Bevan, M.D., Physician to Cook Co. Hospital, has been appointed to the Chair of Public Hygiene in Chicago Medical College. J. P. Ross, M.D., Physician to Cook County Hospital, has been appointed to the Chair of Diseases of the Chest in Rush Medical College.

---

Berkshire Medical College.—This institution, located at Pittsfield, Massachusetts, and for many years prosperous, has finally closed its doors, and will not probably be reöpened.

---

New Medical College.—An effort is being made to organize a new medical college in Detroit, Mich.

---

Michigan University.—It is understood that the Regents of the University of Michigan have abandoned the idea of mixing homœopathy into the medical department of the University.

The Monthly Medical Reprint.—This is a new monthly journal, published by John Hillyer, 14 South William Street, New York. Each number contains 64 closely-printed, double-column pages. It is entirely filled with the reprint "of the most valuable articles published in the latest issues" of the British medical periodicals. Price *five dollars* per annum.

---

The Half-Yearly Abstract of Medical Sciences.—The July No. of this labor-saving volume is promptly on our table, and contains its usual choice selection of foreign medical literature. Published by Henry C. Lea, Philadelphia.

---

University of Nashville.—During the past year, an effort seems to have been made to remove Professors Jennings, Eve, and Jones from their chairs in the Medical Department of the University. They denied the right of the rest of the Faculty to eject them, and appealed to the proper court. A decision has recently been rendered, sustaining their appeal, and enjoining the rest of the Faculty from interfering with them in the performance of their duties in the college.

---

Medical College of the State of South Carolina. Professors Chisolm and Miles have resigned from the chairs which they respectively filled. Professor R. A. Kinloch has been transferred from the chair of materia medica and therapeutics to that of surgery, made vacant by the resignation of Professor Chisolm. Dr. George E. Trescott has been elected professor of materia medica and therapeutics in the place of Professor Kinloch, transferred, and Dr. Middleton Michel has been elected professor of general anatomy and physiology, in the place of Professsor Miles.

---

Medical Department of University of Louisville.—Professors Henry Miller, H. M. Bullit, Lewellyn Powell, Lewis Rogers, and David W. Yandell have resigned their chairs in this Institution. The names of these gentlemen are entirely familiar to the medical public, and the arduous services rendered by most of them have been justly valued and fully appreciated.

The chairs made vacant have been filled by the election of Drs. Theodore S. Bell, John S. Crowe, L. P. Yandell, Jr., and E. R. Palmer, all of Louisville, Ky.

Mortality Report for the Month of June:—

CAUSES OF DEATH.

| | | | | | |
|---|---|---|---|---|---|
| Accident, drowned, | 4 | Enteritis | 1 | Œdema glottitis | 1 |
| " railroad, | 1 | Encephalus, general | 2 | Old Age | 1 |
| " poisoned, | 1 | Erysipelas, | 2 | " and paralysis | 2 |
| Abscess, pelvic | 1 | Exhaustion & old age- | 1 | Paralysis | 2 |
| " hip-joint | 1 | Fever, puerperal | 2 | Peritonitis | 1 |
| Aneurism, rupture. | 1 | " scarlet | 8 | Pleurisy | 1 |
| Apoplexy | 2 | " typhoid | 8 | Pneumonia, | 21 |
| Asphyxia | 1 | " typhus | 1 | Pneumonia, broncho | 2 |
| Asthma | 3 | " pernicious | 1 | Pneumonia, typhoid | 2 |
| Birth, premature | 15 | Gangrene, seniles | 1 | Pneumonia, tuberculus | 1 |
| " still | 32 | " fol. amputa- | | Pneumonia, complicated | |
| Bronchitis | 2 | tion of thigh | 1 | with Measles | 1 |
| " chronic | 1 | Groin, carcinoma of | 1 | Phthisis pulmonalisis | 22 |
| Brain, congestion of | 5 | Hepatitis, chronic | 1 | Phthisis Mesenterica | 1 |
| " disease of | 1 | Heart, disease of | 1 | Scrofula, with fatty de- | |
| " inflammation | 1 | " valvular disease | 2 | generation of liver | 1 |
| Bright's disease | 1 | Hydrothorax, | 2 | Spine, injury of | 1 |
| Bowels, inflammation, | 1 | Hydrocephalus | 1 | Stomach, cancer of | 2 |
| Cancer | 1 | " acute | 3 | " chronic derange. | 1 |
| " of intestines | 1 | Inanition | 5 | Suicide | 4 |
| " of tongue | 1 | Intestines, ulceration- | 1 | Sunstroke | 4 |
| Convulsions | 38 | Kidneys and Liver, | | Small-pox | 9 |
| " puerperal, | 3 | organic disease of | 1 | " complicated | |
| " fm teething | 5 | Kidneys, disease of | 1 | with meningitis | 1 |
| Cholera infantum | 8 | Laryngitis | 2 | Tabes mesenterica, | 6 |
| Chicken-pox, retroces. | 1 | Liver, inflammation of | 2 | Tetanus traumaticus, | 1 |
| Croup | 7 | Lungs, congestion of | 1 | Tetanus | 1 |
| " foll. whoop-cough | 1 | Lungs, paralysis of | 1 | Teething | 2 |
| Debility | 8 | Measles | 9 | " and convulsions, | 2 |
| " and old age, | 1 | " complicated with | | Throat, inflammation | 1 |
| Diffuse inftm. cellular | | pneumonia | 1 | Tumor, abdominal | 1 |
| tissue | 1 | " " bronchitis | 1 | Uterus, cancer of | 3 |
| Delirium tremens | 2 | Meningitis | 1 | Uterus, disease of | 1 |
| Diphtheria | 1 | " cerebro-spinal, | 2 | Uterus, hemorrhage of | 1 |
| Diarrhœa | 13 | " tuberculosis, | 2 | Whooping-Cough | 6 |
| " chronic, | 1 | Mouth, canker sore | 2 | Wounds, inflicted by | |
| Dropsy | 2 | Manslaughter, | 1 | parents, | 1 |
| Dysentery, | 3 | Nephritis | 1 | Total | 305 |

COMPARISON.

Deaths in June, 1868, 305 | Deaths in June, 1867, 283 | Increase, 22

Deaths in May, 1868, 321 | Decrease, 16

AGES.

| | | | | | |
|---|---|---|---|---|---|
| Under 5 | 173 | 40 to 50 | 13 | 90 to 100 | 0 |
| 5 to 10 | 12 | 50 to 60 | 19 | 100 to 110 | 0 |
| 10 to 20 | 11 | 60 to 70 | 19 | Unknown | 2 |
| 20 to 30 | 21 | 70 to 80 | 8 | | |
| 30 to 40 | 23 | 80 to 90 | 4 | Total | 305 |

| | | | | | |
|---|---|---|---|---|---|
| Males, | 173 | Females, | 132 | Total, | 305 |
| Single, | 222 | Married, | 83 | Total, | 305 |
| White, | 298 | Colored, | 7 | Total, | 305 |

NATIVITY.

| | | | | | |
|---|---|---|---|---|---|
| Chicago | 136 | England | 4 | Scotland | 2 |
| Other parts U. S. | 52 | Germany | 42 | Sweden | 14 |
| Africa, | 1 | Holland | 1 | West Indies, | 1 |
| Bohemia | 1 | Ireland | 33 | Unknown | 3 |
| Canada | 3 | Norway | 7 | | |
| Denmark | 2 | Poland | 1 | Total | 305 |

DEATHS BY SMALL-POX.

For the Month of June, 1868.

| | | | |
|---|---|---|---|
| 3d Ward | 2 | 14th Ward | 1 |
| 7th Ward | 0 | Lake Hospital | 3 |
| 12th Ward | 1 | Immigrant | 1 |
| 13th Ward | 2 | | |
| | | Total, | 10 |

MORTALITY BY WARDS FOR THE MONTH.

| Ward. | Mortality. | Pop. in 1868. | One death in | Ward. | Mortality. | Pop. in 1868. | One death in |
|---|---|---|---|---|---|---|---|
| 1 | 2 | 11,499 | 5,749 1-2 | 14 | 14 | 14,168 | 1,012 |
| 2 | 15 | 13,539 | 902 2-3 | 15 | 19 | 20,429 | 1,075 1-5 |
| 3 | 13 | 16,620 | 1,278 1-2 | 16 | 19 | 16,011 | 842 5-8 |
| 4 | 20 | 16,499 | 825 | Unknown, | 1 | | |
| 5 | 16 | 13,434 | 839 2-3 | County hosp. | 7 | | |
| 6 | 17 | 12,407 | 729 7-8 | Chi. River, | 1 | | |
| 7 | 28 | 21,657 | 773 1-2 | Alexian Bros. | 1 | | |
| 8 | 23 | 14,003 | 608 4-5 | Conv't Sa. He. | 1 | | |
| 9 | 12 | 18,050 | 1,504 1-6 | Soldier's hom. | 1 | | |
| 10 | 12 | 13,644 | 1,120 1-3 | Marine hos. | 1 | | |
| 11 | 19 | 13,117 | 690 1-3 | St. Luke's hos. | 1 | | |
| 12 | 11 | 14,739 | 1,340 | Orphan asyl. | 9 | Immigrants, | 23 |
| 13 | 15 | 11,113 | 740 6-7 | Lake Hosp., | 3 | Police Stat. 2d Pre. | 1 |
| Total, | | | | | 305 | | |

MONEY RECEIPTS TO JULY 28TH.—Drs. John Charlton, $3; A. Chapman, 1.50; H. C. Hutchinson, 3; J. M. Steele, 3; S. D. Mercer, 3; J. Y. Campbell, 3; D. C. Stillions, 3; J. W. McAfee, 3.

ON THE NATURE OF VACCINE VIRUS.—The following is an abstract of a paper presented to the Académie des Sciences, by M. A. Chauveau, on the nature of vaccine virus, and the experimental determination of the elements forming the principle of virulent vaccinal serosity:

The virulent humor furnished by the virulent pustule is a complex product, analogous in its composition to all the non-specific pathological serosities. Neither chemical analysis, nor microscopical examinations, have revealed any special element to which the peculiar activity of vaccinia might be attributed. This activity necessarily exists in the common elements which take part in the formation of vaccinal serosity, and which, according to the opinion of Claude Bernard, have acquired a virulent property through simple isomeric modification. But does this metamorphosis, which establishes the virulence, affect all the elements of vaccine? or, rather, does it act upon some one or some few of them; does the virulent activity require the concurrence of all these elements, or is one sufficient for its production? M. Chauveau has attempted to resolve these problems by isolating the principles which enter into the composition of vaccinal serosity, and subjecting each of them to the criterion of physiological experimentation: serum containing, together with albumen which forms the base, all the other soluble substances, was dealt with on the one hand, and on the other, the solid elements, that is to say, the leucocytes and the elementary granular bodies which are suspended in the serous fluid.

M. A. Chauveau derived the following results from his experiments: The leucocytes do not constitute the essential agents of the virulence. They may share in this property with the other elements of vaccinal fluid; but they do not possess it exclusively.

M. Chauveau succeeded in obtaining vaccinal serosity entirely free from all solid bodies, including the finest molecules. This was done by utilizing the phenomenon generally known as diffusion.

Whilst the inoculations of the perfect vaccine succeeded, that of the vaccine deprived of its solid matter always and most completely failed.

M. Chauveau, to give this fact its whole significance, states that "the purely serous liquid was always tested by heat, or nitric acid, at the time of its inoculation, and gave, in every instance, the reaction denoting the presence of albumen. Neither the absence of this fundamental element or of any others, nor their extreme dilution, can, therefore, be brought to explain the inactivity of the vaccinal serous fluid."

These experiments permit of the conclusion that the vaccinal serous fluid is not virulent, and that the activity of the vaccine exists in its solid bodies, either in all without distinction, or in one special portion of these small elementary organisms.

This inactivity of the vaccinal serous fluid, M. Chauveau states, constitutes a fact of great importance, not only with special regard to the theory of virulence, but also from the general point of view of the physiology of the elements.—*Half-Yearly Abstract.*

---

THERMOMETRICAL OBSERVATIONS IN TYPHOID FEVER.—Dr.

Thomson's observations (published in *St. George's Hospital Reports*, vol. ii.) extend over a period of three years, and are made from a careful study of 47 cases. They tend to confirm Professor Wünderlich's researches on the same subject. In typhoid fever, a decrease of temperature is not always a favorable sign, nor is a rapid fall symptomatic only of a crisis. As a rule, the nocturnal increase of temperature in this form of fever is very considerable, and amounts to two or two and a-half degrees. During the latter half of the first week, the heat increases day by day, and varies between 102° and 103° in the morning, and 104° or even 105° in the evening. These high temperatures are quite sufficient to distinguish typhoid fever from tubercular meningitis, or from peritonitis, these diseases seldom showing much increase over 102° Fahr. During the second week, the temperature varies between 102° in the morning, and 103° and 104° in the evening, the oscillations being influenced by the amount of diarrhœa. The thermometer does not enable one to say, by a longer prediction than 24 or 48 hours, whether the case is likely to be fatal or not; but a steady rise in the temperature will often indicate the danger of ulceration of the bowels 24 hours before the intestinal lesion is manifested by diarrhœa and hemorrhage. The mode of termination of the fever is characterized by extraordinary oscillations in the temperature between morning and evening, the difference being sometimes as great as nine degrees. This feature distinguishes the thermograph of typhoid fever from that of almost all other diseases. In one case of perforation of the bowels, which was preceded by hemorrhage, the heat of the body was reduced from 102° to 99°.5, and was followed by a rise to 102°.2, where it remained for 48 hours before death, during which period perforation took place.

The conclusions that the observations made with the thermometer in cases of typhoid fever lead Dr. Thompson to draw, are the following:

1. The thermometer points out a distinction between typhoid fever and some other diseases which often simulate it, *viz.*, acute granular kidney, meningitis, peritonitis.

2. The thermograph of typhoid differs from that of other fevers, and especially in the mode of favorable termination.

3. An additional distinction between typhoid and typhus fevers is thus given.

4. It is possible, by its use, to appreciate intestinal lesions before they are recognized by the ordinary symptoms.—*Half-Yearly Abstract.*

HEMORRHAGE FROM WAXY OR AMYLOID DEGENERATION.—For some years past, Dr. G. T. Stewart, of the Royal Infirmary, Edinburgh, has noticed that hemorrhage from the stomach and intestine occurs in cases of waxy or amyloid degeneration, and that independently of ulceration of the mucous membrane. He has thus been led to look into the literature of the subject, and inquire among professional friends as to their observations.

The conclusions, which seem warrated by the facts he has observed in connection with this subject, are—

1. That hemorrhage is not a very infrequent consequence of the waxy or amyloid degeneration of vessels.
2. That, next to the spleen, the intestinal tract is the most common seat of such hemorrhage.
3. That the hemorrhage occurs independently of any visible ulcerative process.
4. That it probably depends upon rupture of the capillaries at the affected parts.
5. That waxy or amyloid degeneration of the liver does not of itself suffice to induce hemorrhage from the bowels.
6. That the hemorrhage occurs in cases in which the liver is free from waxy degeneration.
7. That the occurrence of hemorrhage increases the danger of the patient. But,
8. That sometimes it comes and goes for years without markedly depressing the vital powers.

In regard to treatment, Dr. Stewart adds, so far as he has yet seen, the diarrhœa and hemorrhage appear to be beter controlled by sedative and astringent enemata than by any other means.—*Ibid*

---

ALCOHOLIC RHEUMATISM.—About 30 years since, John Higginbottom, Esq., F.R.S., Nottingham, first noticed that a form of (so-called) rheumatism was cured by abstaining for some time from the use of fermented alcoholic fluids. He said at that time that the complaint should not be called rheumatism, but alcoholism, as *alcohol* produced the disease, and abstinence from *alcohol* was the remedy. Mr. Higginbottom maintains that:—

1. Alcoholic rheumatism is the result of a distinct cause.
2. It is produced by drinking fermented alcoholic beverages.
3. It is slow in effecting a marked visible change in the system.
4. It does not usually appear before middle life.
5. Its effects are produced by the accumulation of the fermented alcoholic fluids taken into the system.

6. It causes stupidity, stiffness in the body, hobbling gait, and ultimate lameness.

7. It causes changes of structure, producing chronic alcoholism.

8. The remedy is abstinence from the use of all fermented alcoholic drinks, and taking vigorous exercise in the open air. —*Ibid.*

---

A REMARKABLE CASE.—At the late session of the Massachusetts Medical Society, in Boston, Dr. John M. Harlow gave the history of a man named Gage, who, while blasting rocks at Cavendish, Vt., in 1847, had a tamping iron three feet seven inches long and one and a-quarter inch thick and tapering to a point, forced through his head, it entering the left cheek and coming out about the centre of the top of his head. Dr. Harlow, who attended the man, gave the daily symptoms of his patient and said that in 59 days he was able to walk and ride, and was soon nearly as well as before, although his intellect was somewhat affected. This is considered the most remarkable case of the recuperative powers of nature and has been doubted by many prominent surgeons. Gage died May 21, 1861, 12 years, 6 months and 8 days after the injury. Dr. Harlow procured his head and has presented the skull to the Warren museum of the Harvard Medical College. Dr. Bigelow said he saw Gage 20 years ago, and was then satisfied of the reality of this wonderful case. He also said, a tube of iron ⅝ths of an inch in diameter and about five feet long passed through a miner's head, while blasting coal in Ohio, and was pulled out by a fellow-miner. The injured man was introduced to the audience, and Dr. Jewett, the attendant physician, recounted the case in detail.

---

TRANSFUSION OF BLOOD.—A German medical journal gives the account of a case of poisoning by exposure to the vapors of burning charcoal, in which transfusion of blood, from the arm of a robust man, effected a satisfactory cure, after every other effort at restoration had failed and the patient was believed to be dead.

---

EPISTAXIS AND THE MEANS OF ARRESTING IT.—Dr. John Thompson (British Medical Journal—Canada Medical Journal) recommends the following simple and, in his hands, very successful treatment, as a substitute for the ordinary method of plugging the nares. A strip of lint as broad as the finger and twice the length of the nasal passage, is folded over the "bowl end" of an ordinary director and introduced along the floor of the nostril until it reaches the throat. The director is then withdrawn, "giving it a wriggling movement, so as leave the lint rumpled and loosely distending the passage. The result is, that the blood rapidly permeates and distends the lint, a large coagulum is formed and the bleeding completely arrested."—*Humboldt Medical Archives.*

---

